# Supplementary material for: The distribution of fitness effects of spontaneous mutations in Chlamydomonas reinhardtii inferred using frequency changes under experimental evolution
Source: PLoS Genet. 2022 Jun 15;18(6):e1009840. doi: 10.1371/journal.pgen.1009840 (PMC9239454; doi:10.1371/journal.pgen.1009840)
Supplement: S1 Text — (PDF) [file pgen.1009840.s001.pdf]

Text S1

Calculation of scaled recombination rates per chromosome

Let y_i be the scaled recombination rate for chromosome *i*, x_i be its length, and *b* the slope of the linear relationship between recombination rate and map length, i.e.,

$$y_i = bx_i + k \tag{1S}$$

If *n* is the number of chromosomes, the mean recombination rate is:

$$\bar{y} = \frac{\sum_i^n x_i y_i}{\sum_i^n x_i} \tag{2S}$$

Substituting (1S) into (2S), we obtain:

$$k = \bar{y} - b \frac{\sum x_i^2}{\sum x_i}. \tag{3S}$$
