# Supplementary material for: The distribution of fitness effects of spontaneous mutations in Chlamydomonas reinhardtii inferred using frequency changes under experimental evolution
Source: PLoS Genet. 2022 Jun 15;18(6):e1009840. doi: 10.1371/journal.pgen.1009840 (PMC9239454; doi:10.1371/journal.pgen.1009840)
Supplement: S1 Table — (PDF) [file pgen.1009840.s002.pdf]

Table S1. Estimated numbers of generations of experimental evolution.

Recombinant population	Number of days of experimental evolution	Number of generations replicate 1	Number of generations replicate 2	Number of generations replicate 3	Mean number of generations	SD of number of generations
L06	42	58.33	57.92	57.96	58.07	0.2257
L09	42	58.79	58.90	59.15	58.96	0.1879
L10	46	59.95	59.38	60.80	60.05	0.7156
L12	46	61.03	60.60	59.70	60.44	0.6766
L13	46	59.40	60.02	60.94	60.12	0.7739
L14	46	60.61	60.81	60.22	60.55	0.2968
L15	46	60.10	61.32	60.61	60.67	0.6121
