# Supplementary material for: The distribution of fitness effects of spontaneous mutations in Chlamydomonas reinhardtii inferred using frequency changes under experimental evolution
Source: PLoS Genet. 2022 Jun 15;18(6):e1009840. doi: 10.1371/journal.pgen.1009840 (PMC9239454; doi:10.1371/journal.pgen.1009840)
Supplement: S2 Table — (PDF) [file pgen.1009840.s003.pdf]

Table S2. Difference in effect sizes and effect sizes squared between annotation categories along with P-values obtained by bootstrapping by mutation 10,000 times.

**Annotation type contrast**	**Mean effect difference (x1000)**	**P-value**	**Mean squared effect difference (x1000)**	**P-value**
Exonic v non-exonic	-0.00316	0.91	0.000038	0.77
Genic v non-genic	0.00851	0.89	0.000009	0.78
Nonsynonymous v synonymous	-0.0110	0.77	0.000594	0.71
