# Supplementary figures and images for: The distribution of fitness effects of spontaneous mutations in Chlamydomonas reinhardtii inferred using frequency changes under experimental evolution
Source: PLoS Genet. 2022 Jun 15;18(6):e1009840. doi: 10.1371/journal.pgen.1009840 (PMC9239454; doi:10.1371/journal.pgen.1009840)

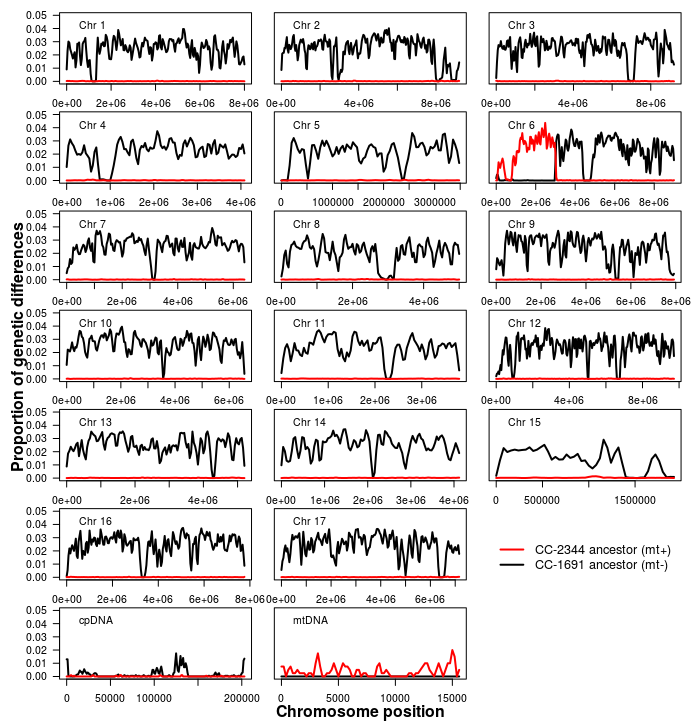

Supplement: S1 Fig — The proportion of genetic differences for 80-kb windows in the case of the chromosomes and for 2-kb and 200-bp windows for the cpDNA and mtDNA, respectively, were calculated based on variant tables extracted from the VCF file using the VariantsToTable tool of GATK [22,23]. (TIF) [file pgen.1009840.s004.tif]

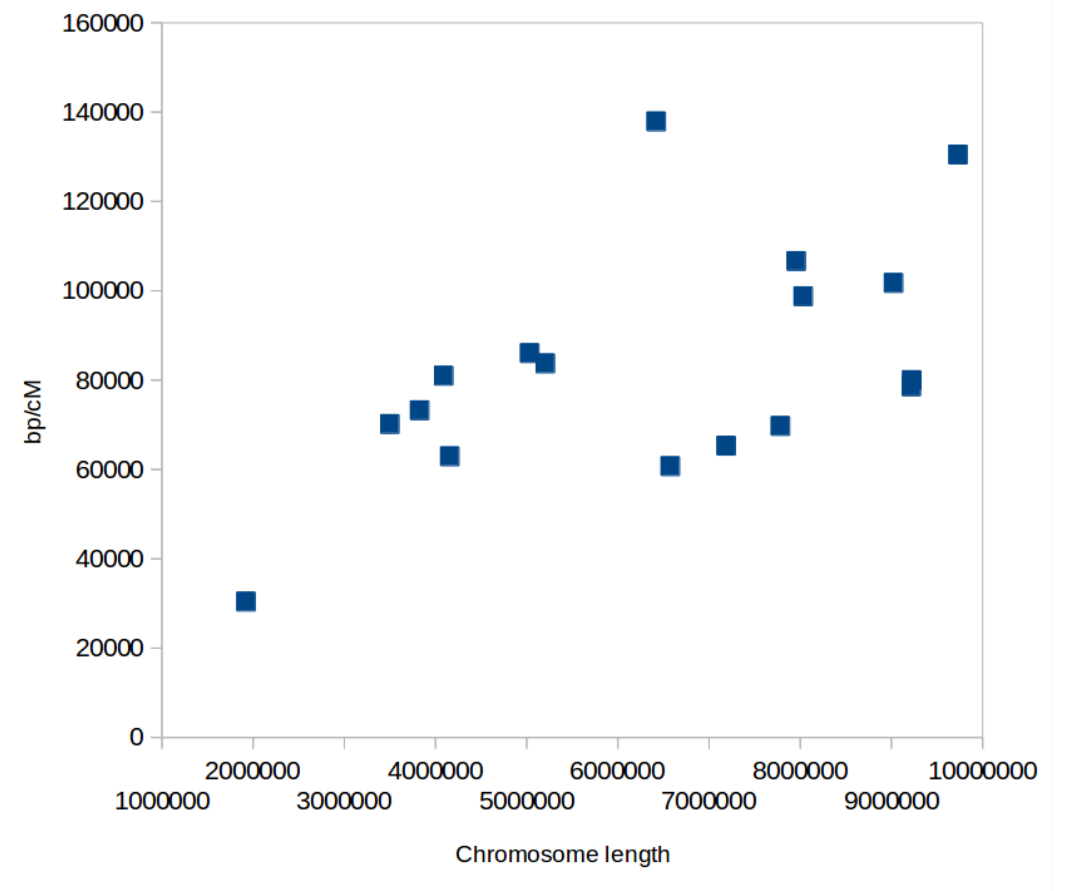

Supplement: S2 Fig — (Pearson correlation r = 0.56; linear regression: bp/cM = 0.00616 x length + 43,900, P = 0.015). (PNG) [file pgen.1009840.s005.png]

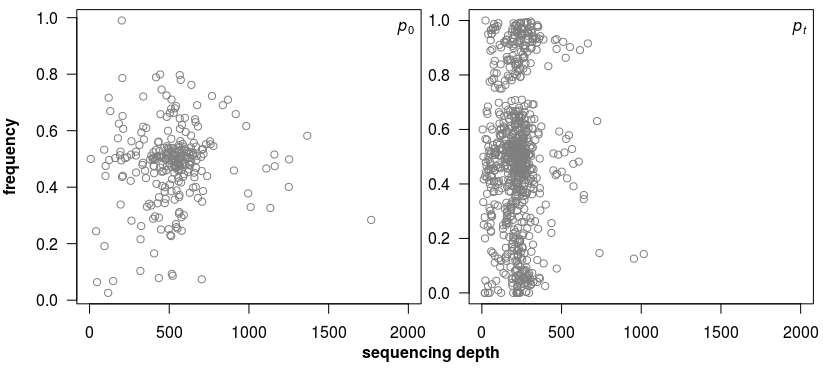

Supplement: S3 Fig — Mean sequencing depth for the mutations are 520.7x and 221.6x for times 0 and t, respectively. (TIF) [file pgen.1009840.s006.tif]

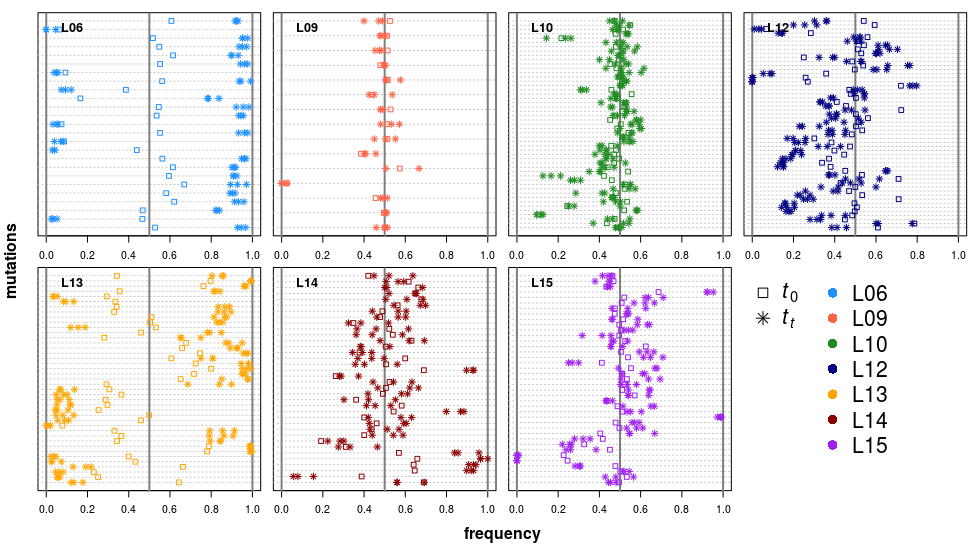

Supplement: S4 Fig — Mutations of each MA line are shown from top to bottom in the order in which they occur in the genome. Squares denote the mutation frequencies at t0, and stars denote the mutation frequencies of the three replicates at tt. The different MA line recombinant pools are shown in the different panels. (TIF) [file pgen.1009840.s007.tif]

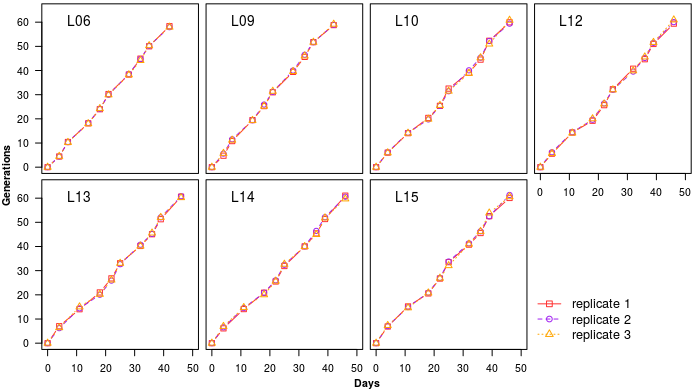

Supplement: S5 Fig — Each panel shows the cumulative number of generations of the three replicates of each of the seven recombinant populations derived from a backcross between MA line and compatible ancestor. Number of generations was estimated from OD measurements conducted at each transfer. (TIF) [file pgen.1009840.s008.tif]

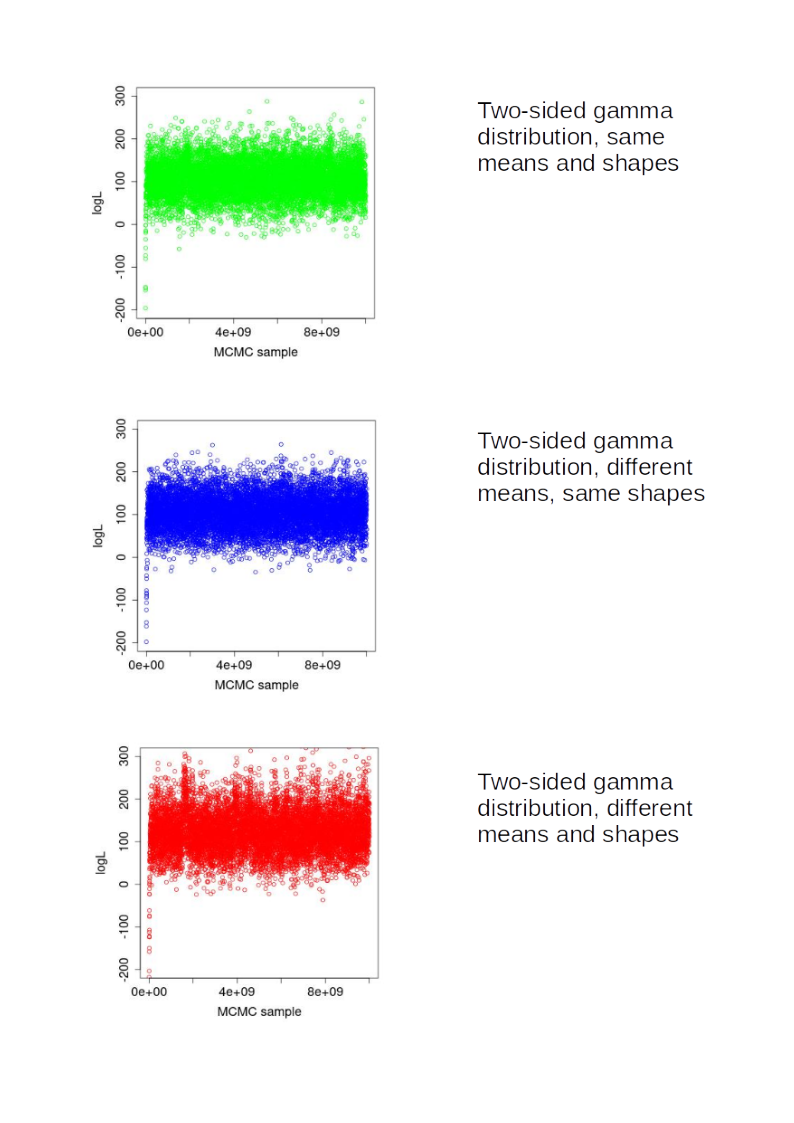

Supplement: S6 Fig — (TIF) [file pgen.1009840.s009.tif]
